# From Cell to Beak: In-Vitro and In-Vivo Characterization of Chicken Bitter Taste Thresholds

**DOI:** 10.3390/molecules22050821

**Published:** 2017-05-17

**Authors:** Shira Cheled-Shoval, Maik Behrens, Ayelet Korb, Antonella Di Pizio, Wolfgang Meyerhof, Zehava Uni, Masha Y. Niv

**Affiliations:** 1 Department of Animal Science, The Robert H. Smith Faculty of Agriculture, Food, and Environment, The Hebrew University of Jerusalem, Rehovot 76100, Israel; Shira.cheled@gmail.com (S.C.-S.); ayelet.korb@gmail.com (A.K.); zehava.uni@mail.huji.ac.il (Z.U.); 2 The Institute of Biochemistry, Food Science and Nutrition, The Robert H. Smith Faculty of Agriculture, Food, and Environment, The Hebrew University of Jerusalem, Rehovot 76100, Israel; antonelladipizio@gmail.com; 3 Department of Molecular Genetics, German Institute of Human Nutrition Potsdam-Rehbruecke, 14558 Nuthetal, Germany; behrens@dife.de (M.B.); meyerhof@dife.de (W.M.)

**Keywords:** bitter taste, chicken, threshold, in-vivo, in-vitro, avian, Tas2r, T2R, ggTas2r, calcium imaging

## Abstract

Bitter taste elicits an aversive reaction, and is believed to protect against consuming poisons. Bitter molecules are detected by the Tas2r family of G-protein-coupled receptors, with a species-dependent number of subtypes. Chickens demonstrate bitter taste sensitivity despite having only three bitter taste receptors—ggTas2r1, ggTas2r2 and ggTas2r7. This minimalistic bitter taste system in chickens was used to determine relationships between in-vitro (measured in heterologous systems) and in-vivo (behavioral) detection thresholds. ggTas2r-selective ligands, nicotine (ggTas2r1), caffeine (ggTas2r2), erythromycin and (+)-catechin (ggTas2r7), and the Tas2r-promiscuous ligand quinine (all three ggTas2rs) were studied. Ligands of the same receptor had different in-vivo:in-vitro ratios, and the ggTas2r-promiscuous ligand did not exhibit lower in-vivo:in-vitro ratios than ggTas2r-selective ligands. In-vivo thresholds were similar or up to two orders of magnitude higher than the in-vitro ones.

## 1. Introduction

Bitter taste perception is involved in detecting potentially toxic compounds [1,2,3]. In vertebrates, bitter taste recognition is mediated by the family of bitter taste receptors (the Tas2r family) and their downstream signaling components [4]. The number of bitter taste receptor subtypes varies widely, and ranges from none in penguins to ~80 in the coelacanths [5,6,7,8,9,10,11,12]. In chicken, a low to non-existing sense of taste was previously assumed [13,14]. This assumption was based mainly on the lower numbers of taste buds in mature chickens compared to humans, 250–760 vs. ~9000, respectively [15]. Nevertheless, 69% of chicken taste buds are located on the upper palate, 29% on the lower mandible and only 2% on the posterior part of the tongue [16], which may lead to false conclusions when examining taste-bud counts on chickens’ tongues. However, in the last few years, insights from morphological, behavioral, molecular and comparative studies have demonstrated chickens have a developed sense of taste, disproving earlier assumptions of lower taste acuity [14].

Indeed, both gustatory and extra-gustatory mechanisms involving taste signaling have been recently shown in chickens [7,14,17,18,19,20,21,22,23,24], suggesting important applications of chemoreception in these animals. Thus, the identification of bitter tastants and their detection thresholds is important for studying potential effects on chicken feeding behavior. Interestingly, chickens have only three bitter taste receptor genes in their genome: *ggTas2r1*, *ggTas2r2* and *ggTas2r7* [25]. The low number of bitter taste receptors makes the chicken a valuable minimalistic model for an understanding of vertebrate taste perception. We demonstrated the expression of the three bitter taste receptors and downstream signaling genes in the chicken oral cavity and gastrointestinal tract [18]. Cheled-Shoval et al. [26] used in-vivo threshold tests to elucidate chicken detection thresholds for bitter, umami and sweet representative tastants. Behrens et al. [7] reported that based on functional expression assays, all three chicken bitter taste receptors are broadly tuned, meaning that a large average number of ligands per receptor compensates for the low number of receptors. The information on ligands that activate ggTas2rs was incorporated into BitterDB [27], a publicly available database of bitter compounds. The selectivity and promiscuity of bitter compounds and their receptors were further analyzed [28], suggesting that promiscuity profiles of bitter ligands toward Tas2rs are conserved across species. Furthermore, some ligands are Tas2r-selective, while others activate multiple Tas2rs [27,28]. Here we investigate the relation between in-vivo to in-vitro detection thresholds. The fact that there are only three Tas2r subtypes in chicken makes it possible to answer the following questions: What is the typical relationship between in-vivo and in-vitro thresholds? Is there a difference between Tas2r-selective and Tas2r-promiscuous ligands? Answering these questions will help filling the gap between behavioral studies and molecular and functional assays. In the current study, the relations between in-vivo and in-vitro thresholds are explored using the minimalistic chicken Tas2r system.

## 2. Results

### 2.1. All ggTas2rs Are Expressed in Transfected Cells

First, expression of the chicken Tas2r constructs in HEK 293T-Gα16gust44 cells was established by immunocytochemical analysis. Whereas Tas2r2 and Tas2r7 were expressed at 40.0 ± 5.0% and 43.9 ± 7.8% of the cells, respectively, the receptor cDNA of Tas2r1 exhibited somewhat lower expression, 21.9 ± 3.5%. Thus, expression of the receptors in the transfected cells was confirmed. There was no evident difference in the receptors’ cell-surface localization (see Supplementary Data and Figure S1).

### 2.2. Bitter Compounds Show Different In-Vitro Detection Thresholds

**Figure 1:**
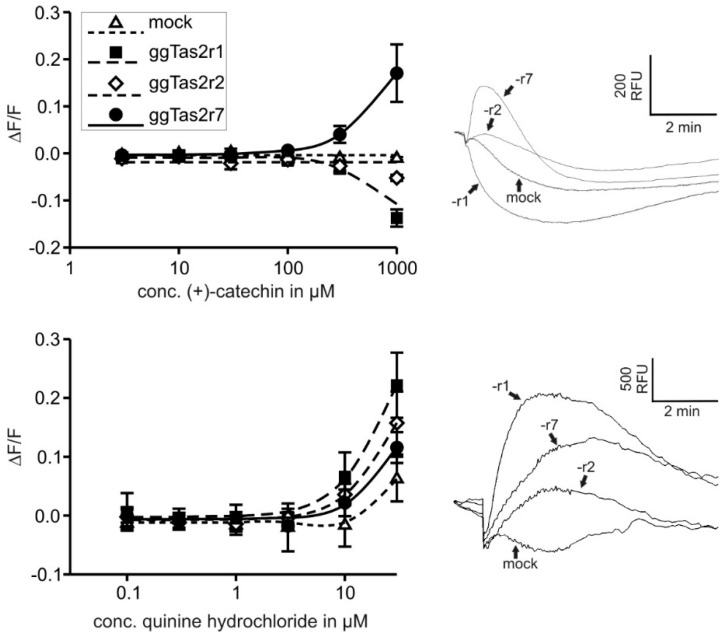
Functional analyses of chicken Tas2rs. The cDNAs of the three chicken Tas2rs were transiently transfected in HEK 293T-Gα16gust44 cells and challenged with different concentrations of (+)-catechin (upper panel) and quinine hydrochloride (lower panel). The changes in fluorescence (ΔF/F) were monitored and plotted against the log compound concentration (*x*-axis). Raw calcium traces obtained with 1000 µM (+)-catechin (upper right panel) and 30 µM quinine hydrochloride (lower right panel), respectively, are diplayed next to the corresponding dose-response curves (scale: *y*-axis, 500 relative fluorescence units (RFU); *x*-axis, time in minutes (max 2 min)) r1 = ggTas2r1; r2 = ggTas2r2; r7 = ggTas2r7; Mock = empty plasmid which represents negative control).

Table 1 summarizes the newly obtained (+)-catechin and quinine hydrochloride (QH) results shown in Figure 1 and lists previously determined in-vitro thresholds for several additional compounds [7]. Interestingly, single-receptor agonists (nicotine, caffeine, erythromycin and the newly discovered (+)-catechin) demonstrate in-vitro thresholds of the same magnitude (0.1 mM to 0.3 mM), whereas QH, the multi-receptor agonist, has a one order of magnitude lower in-vitro threshold (0.01 mM for all receptors).

### 2.3. In-Vivo Avoidance Thresholds

#### 2.3.1. Nicotine

**Figure 2:**
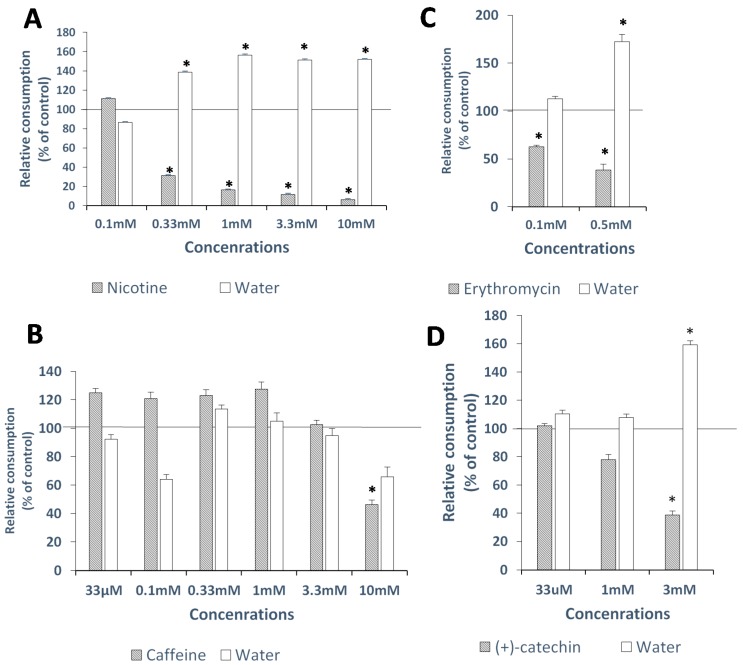
Effects of different concentrations of bitter tastants on consumption parameters. Tastant side consumption (filled bars: (**A**) Nicotine; (**B**) Caffeine; (**C**) Erythromycin; (**D**) (+)-Catechin) and water side consumption per chick (open bars) as percentage of control, during 24 h. Consumption parameters were normalized to the distilled water control group (=100%, indicated by black line at 100). Bars represent consumption (represented as percentage of control group) ±SEM. * Significantly different (*p* ≤ 0.05) from control group using Dunnett’s Method.

At 0.33 mM and up, both the nicotine consumption parameter and the proportion of nicotine consumption out of total consumption (ratio) were significantly decreased relative to the control (*p* < 0.0001 for both). In addition, water consumption was significantly higher than in the control group (*p* = 0.0027) (Figure 2A). See Supplementary Figure S2A for total consumption and ratio parameters.

#### 2.3.2. Caffeine

At 10 mM, caffeine consumption and total consumption were significantly decreased as compared to the control (*p* = 0.0347 and 0.0005, respectively). No significant changes were found in the other two consumption parameters at 10 mM (Figure 2B and Figure S2B).

#### 2.3.3. Erythromycin

At 0.1 mM and 0.5 mM, erythromycin consumption and the ratio parameter were significantly decreased compared to controls (*p* = 0.0004, *p* < 0.0001 and *p* = 0.0031, *p* < 0.0001, respectively). At 0.5 mM erythromycin, water consumption was significantly higher than in the control group (*p* < 0.0001) (Figure 2C and Figure S2C).

#### 2.3.4. (+)-Catechin

At 3 mM, both (+)-catechin consumption and the ratio parameter were significantly decreased compared to the control group (*p* = 0.0015 and 0.0011, respectively), while water consumption was significantly higher than in the control group (*p* = 0.0047) (Figure 2D and Figure S2D). For QH, an in-vivo threshold of 0.3 mM has been already reported previously [26].

### 2.4. ggTas2rs Exhibit Different Expression Levels in the Palate

**Figure 3:**
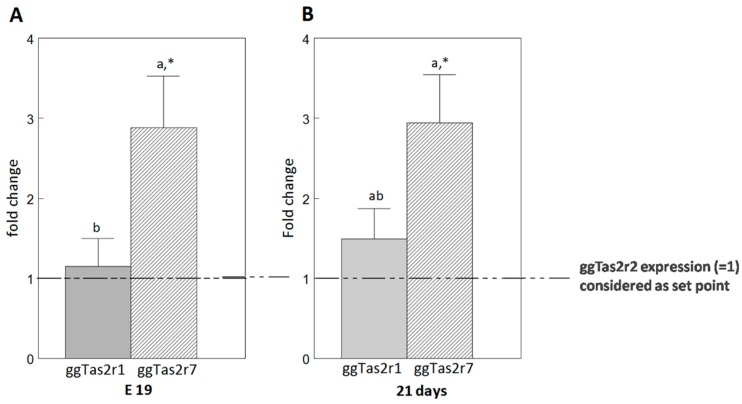
Relative mRNA abundance of ggTas2r-encoding genes (*ggTas2r1*, *ggTas2r2*, *ggTas2r7*) on E19 (**A**) and at 21 days (**B**) with the *ggTas2r2* serving as the set-point gene (relative expression set to 1, n = 6), obtained from data published in Cheled-Shoval et al. [18]. Values are presented as mean fold change ±SEM. Differences among the *ggTas2r1* and *ggTas2r7* genes within the palate: means without a common letter differ significantly (*p* < 0.05); differences between the tested genes (*ggTas2r1* and *ggTas2r7*) and the control gene (*ggTas2r2*) within the palate: means with an asterisk (*) differ significantly from *ggTas2r2*.

To relate the in-vivo to in-vitro thresholds, the expression levels of the receptors in the palate should be taken into consideration. We analyzed raw expression data that was obtained in Cheled-Shoval et al. [18] in order to compare the levels of gene expression of the three *ggTas2r*s in E19 (embryonic day 19) (Figure 3A) and 21-day-old (Figure 3B) broiler chickens’ upper palate, the main location of taste buds in the chicken oral cavity [16,18]. *ggTas2r7* expression level is significantly higher than *ggTas2r1* and *ggTas2r2* expression levels in the embryo (E19). In the growing broiler (21d) *ggTas2r7* expression is significantly higher than *ggTas2r2*, but not significantly different from *ggTas2r1* expression level.

### 2.5. Variation in In-Vivo:In-Vitro Ratios

The in-vivo avoidance threshold for caffeine (ggTas2r2-specific) was ~30-times higher than in-vitro (10 mM in-vivo vs. 0.3 mM in-vitro). This could have been attributed to the lower expression levels of the caffeine-cognate receptor gene *ggTas2r2* (in comparison to *ggTas2r7*). However, QH, which activates all three ggTas2rs, also demonstrated a 30-times increase for the in-vivo avoidance vs. in-vitro detection threshold (0.3 mM and 0.01 mM, respectively).

**Figure 4:**
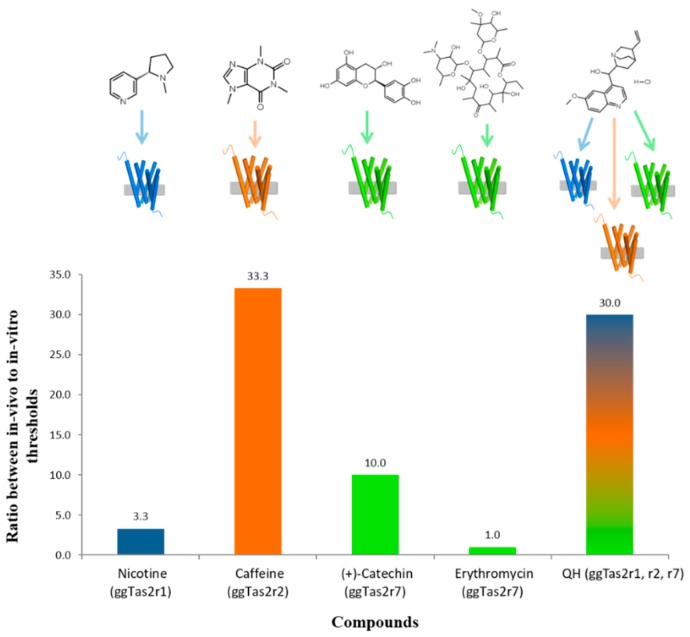
Comparison of in-vivo and in-vitro thresholds. Blue, orange and green colors represent the in-vivo:in-vitro ratios for ggTas2r1, ggTas2r2 and ggTas2r7, respectively.

The ggTas2r1-specific ligand nicotine demonstrated similar in-vivo and in-vitro thresholds (0.33 mM and 0.1 mM, respectively), despite expression levels similar to *ggTas2r2*. Furthermore, ligands activating only ggTas2r7, differed in the in-vivo:in-vitro ratios: (+)-catechin showed a 10-times increase for the in-vivo avoidance threshold (3 mM) compared to the in-vitro one (0.3 mM), while erythromycin had the lowest thresholds, which were similar for in-vivo avoidance behavior and in-vitro receptor responses (both at 0.1 mM). The in-vitro and in-vivo thresholds concentrations and their ratios are summarized in Table 1 and Figure 4.

## 3. Discussion

Chicken taste research has two main directions: the first addresses agricultural issues in an effort to understand taste perception in chickens and affect feeding behavior, intake and performance resulting from taste cues [17,18,20,21,22,23,24]. The second addresses comparative biology issues using the chicken as a model for uncovering vertebrate taste-perception mechanisms [7,14,27,29,30].

In this study, we used the minimalistic Tas2r system in chicken to explore the relations between in-vitro and in-vivo thresholds for different bitter ligands, including the newly identified (+)-catechin. The ligands varied in their in-vivo:in-vitro threshold ratios and there were no apparent relations between the ligand characteristics (selectivity or promiscuity) and the subtype of ggTas2r activated with the in-vivo-avoidance or in-vitro-detection threshold concentrations.

### 3.1. Relationships between In-Vivo and In-Vitro Thresholds

A comparison of in-vivo and in-vitro concentration thresholds suggests that, for the examined bitterants, there is no simple relationship. Even same-receptor activators with similar in-vitro thresholds ((+)-catechin and erythromycin for ggTas2r7) had different in-vivo avoidance thresholds. The number of receptors activated in-vitro also could not explain the in-vivo thresholds: though one might expect that a ligand that activates multiple bitter taste receptors in-vitro, may be more aversive in-vivo than ligands that activate a single receptor, such a trend was not found. Specifically, single-receptor agonists tested—erythromycin, nicotine, caffeine and (+)-catechin—demonstrated higher, similar or lower (respectively) in-vivo thresholds than the promiscuous agonist QH.

In-vivo vs. in-vitro bitter-tastant threshold comparison in chickens, reported by Hirose et al., showed identical in-vivo and in-vitro threshold concentrations for the bitter tastants dextromethorphan and diphenidol [19]. Very recently, Dey et al. [31] have performed in-vivo tests of several ggTas2r2- and ggTas2r7-specific agonists. Interestingly, ggTas2r2-specific agonists tested in that research, including caffeine, did not show significant intake differences compared to water. Notably, the highest concentration of caffeine tested by Dey et al. (3 mM) was lower than the threshold identified here. In these studies, the no-choice in-vivo tests probably elevated the in-vivo threshold compared to the two-choice method used here, due to the nature of the test i.e., the chicks are under restricted water regime for 6 h prior to the test, which may cause them to consume the drinking solution despite its bitter taste. In addition, the chicks in these studies were of the layers Rhode Island Red strain, which was shown to be less sensitive to bitter taste than broilers [21].

Differences between in-vivo and in-vitro thresholds have also been reported in humans. For example, in-vivo thresholds for caffeine have been reported to be between 1 and 5 mM [32], whereas its in-vitro threshold is reported to be 0.3 mM [3], a ratio of 3–16 for in-vivo:in-vitro thresholds. Similarly, the in-vivo threshold concentration reported for quinine is ~12 times higher than its in-vitro threshold concentration (0.12 mM vs. 0.01 mM) [3,33,34]. In addition, similarly to chicken, no relation between the number of receptors activated and in-vivo thresholds was found in humans; quinine and caffeine, both multiple-receptor activators (9 Tas2r and 5 Tas2rs, respectively), showed higher in-vivo thresholds than phenylthiocarbamide (PTC), a single-receptor activator [3,32,34,35].

### 3.2. Possible Reasons for Differences between In-Vivo and In-Vitro Thresholds

#### 3.2.1. Avoidance vs. Recognition

Avoidance behavior does not necessarily tightly correlate with genuine recognition thresholds, as some species tolerate and consume bitter substances at supra-threshold concentrations [36]. Development of taste recognition (rather than avoidance) paradigm for chickens presents an interesting future challenge.

#### 3.2.2. Different Expression Levels of the Cognate Receptors

The number of taste buds in chicken peaks at E19 and remains relatively constant thereafter [37]. The expression of *ggTas2r7* was significantly higher than of *ggTas2r1* and *ggTas2r2* on E19 and of *ggTas2r2* at 21 days. The expression results obtained by real-time PCR do not enable concluding on whether the significantly low expression seen in *ggTas2r2* represents many cells with low mRNA expression or on the contrary, a few cells with high mRNA expression; both may affect in-vivo thresholds. However, it is clear that different expression levels do not explain the variation between in-vivo vs. in-vitro thresholds, as discussed in the Results section on variations in in-vivo:in-vitro ratios.

#### 3.2.3. Additional Sensory Properties

Some compounds, such as nicotine, may possess additional sensory properties, such as olfactory or trigeminal components for rodents [38]. The sensory properties of the tested ligands have not yet been established for chickens, but the fact that in-vivo threshold is equal or higher than the in-vitro ggTas2r threshold, suggests that the potential additional sensory properties do not dominate the aversion effect.

#### 3.2.4. Test Length

The length of the tests (24 h) may also be a factor affecting the in-vivo responses to the compounds via physiological effects that may affect the chicks’ drinking behavior. The longer the test, the higher the potential impact of postingestive effects. In the literature, in-vivo behavioral tests in chickens varied from 10 min to 33 days. It should be noted that most molecules have biological effects, ranging from a few min after consumption and up to hours and days, which can affect behavioral responses. Shorter tests periods have been tested by our group and others [19,21,26,31], but the results of the tests are significantly less sensitive for threshold determination [21,26]. A comprehensive discussion on the effect of test duration can be found in Cheled-Shoval et al. [26].

Notably, gustatory mixtures may create a complex effect, as different tastants can either raise or decrease the detection thresholds of each other [39]. In addition, the interaction of different taste modalities tastants with volatile flavor elements may contribute to the complexity of taste perception [40]. Studies in humans show that interactions between different tastants depend on their concentrations, the properties of the media (feed/drink) and the experimental methods used [40,41,42]. The minimalistic gustatory system in chicken provides a convenient model to study the effects of mixing several bitter compounds, combining bitter tastants with other taste modalities and changing the delivery medium (liquid vs. solid).

To conclude, one of the major gaps in taste-related animal research is relating molecular or physiological methods to behavior. The results of this study indicate that even in the simplest possible Tas2r system such as chicken, the in-vitro and in-vivo relations are complicated and the strength of the in-vivo response could not be accurately deduced from cellular data. However, the current results show that the in-vivo thresholds are typically similar or up to two orders of magnitude higher than the in-vitro ones and that, on a qualitative level, in-vitro activation of chicken bitter taste receptors predicts aversive behavior to these compounds in-vivo.

## 4. Materials and Methords

### 4.1. Chemicals

All compounds were obtained from Sigma-Aldrich (Taufkirchen, Germany or Rehovot, Israel): (−)-nicotine (C_10_H_14_N_2_, #36733), erythromycin (C_37_H_67_NO_13_, #E5389), caffeine (C_8_H_10_N_4_O_2,_ #C0750), (+)-catechin hydrate (C_15_H_14_O_6_·2H_2_O, #C1251), and quinine hydrochloride dehydrate (C_20_H_24_N_2_O_2_·HCl·2H_2_O, #Q1125).

#### 4.1.1. Functional Expression of Chicken Tas2r Constructs

The functional expression of chicken Tas2r constructs was performed as published previously [7,17]. Briefly, HEK 293T-Gα16gust44 cells were seeded in 96-well plates and transiently transfected with constructs encoding ggTas2r1, ggTas2r2 or ggTas2r7, as well as with the empty expression vector pcDNA5FRT. Twenty-four hours after transfection, the cells were loaded with the fluorescent dye Fluo4-AM in the presence of 2.5 mM probenecid and placed in a fluorometric imaging plate reader (FLPR-Tetra, Molecular Devices, Sunnyvale, CA, USA). Cells transfected with empty vector (mock) served as negative control. After automatic application of different concentrations of the test substances (+)-catechin and QH, changes in fluorescence were monitored and used for the calculation of dose-response relationships using SigmaPlot software (v12.3, Erkrath, Germany), as described previously [7,17].

#### 4.1.2. Immunocytochemistry

Cloning of the three chicken cDNAs in the vector pcDNA5FRT was as detailed previously [7,17]. More details can be found in the Supplementary Data.

### 4.2. Taste Tests

#### 4.2.1. Animals and Maintenance Conditions

We acquired 1-day-old chicks (Cobb 500) from a maternal flock (Brown Ltd., Hod Hasharon, Israel). All chicks were placed in separate pens (40 × 40 cm) in a brooding house (3 birds/pen). The chick feed, which was formulated to meet National Research Council (1994) requirements as appropriate, was offered *ad libitum*. Water was freely available until tests were performed. All experimental procedures followed established guidelines for animal care and handling, and were approved by the Hebrew University Institutional Animal Care and Use Committee (AG-13196).

#### 4.2.2. In-Vivo Avoidance Threshold Detection Tests

All taste tests were conducted as described previously [26]. Briefly, two-alternative forced-choice (2-AFC) tests comprised of two to six ascending concentrations of the tastants (Table 2).

A separate group of chicks was used for each tastant—for nicotine, n = 90, for caffeine, n = 105, for erythromycin, n = 45, and for (+)-catechin, n = 60. Chicks were divided into equal-weight groups (one treatment group per concentration and one control group—water only). Each treatment group (one concentration) was further divided into five replications (three chicks/replicate). After a three days’ adaptation period, two 250 mL bottles (equipped with drinking nipples by Plasson^®^ Israel, (Maagan Michael, Israel)) were filled with a tastant solution or water, weighed and placed 20 cm apart on each side of the back of each pen, opposite the feed. At the end of the 24 h trial, consumption from the two bottles was measured by weighing. Consumption results were expressed as four measurement parameters: (1) consumption from the tastant solution side; (2) consumption from the water side; (3) total consumption (from both the water and tastant solution sides) and (4) ratio of consumption on the tastant solution side to total consumption. The lowest concentration (of each tastant) causing a significant change in one or more of the four measured parameters was defined as the tastant-detection threshold. The tastant solution was placed randomly, following a recent study [26].

#### 4.2.3. mRNA Abundance (Fold Change) Calculation Using the Comparative ΔΔCt Method

All raw mRNA abundance data [18] were subjected to new ΔΔCt fold-change calculations [43], and statistical analysis was performed to compare the expression of the three *ggTas2r*s in the palate. Briefly, the efficiencies of all of the tested genes and housekeeping genes were calculated. Cycle threshold (Ct) values for each sample were calculated using StepOne software v 2.1 (Applied Biosystems, Foster City, CA, USA) and gene expression was normalized against the geometric average of *β-actin* and *Cyc A*. Changes in mRNA abundance were analyzed by comparing the relative expression among the genes in the palates. To compare genes, *ggTas2r2* was chosen as the set-point gene for the ΔΔCt analysis and fold changes were calculated relative to the level of this chosen gene (i.e., *ggTas2r2* gene expression was set to 1), as described in detail in previous publications [18,44]. Primers and genes accession numbers are described in Table S1 in the Supplementary Data.

### 4.3. Statistical Analysis

#### 4.3.1. Taste Tests

These were performed as described previously [26]. One-way ANOVA was run and differences between means of treatment groups and the control group were calculated using Dunnett’s test (marked with *).

#### 4.3.2. Gene Expression

For gene-expression analyses, relative expression of the genes *ggTas2r1* and *ggTas2r7* was compared to the chosen gene (*ggTas2r2*) using ANOVA, and differences between means of *ggTas2r1* or *ggTas2r7* and the gene *ggTas2r2* were calculated using Dunnett’s test (marked with *). In addition, differences between *ggTas2r1* and *ggTas2r7* means were calculated using Tukey–Kramer HSD and significantly different means are indicated by different letters (a, b). An alpha level of 0.05 was used for all tests. All statistical analyses were conducted with JMP Pro 12 software (SAS Institute, Cary, NC, USA, 2006).

## Figures and Tables

**Table 1:** In-Vitro and in-Vivo Avoidance Thresholds for Chicken.

Compound	Threshold Concentrations (mM)		Receptor Activated In-Vitro
	In-Vitro	In-Vivo	ggTas2r1
Nicotine	0.1 (Behrens et al. [7])	0.33	**ggTas2r2**
Caffeine	0.3 (Behrens et al. [7])	10	**ggTas2r7**
Erythromycin	0.1 (Behrens et al. [7]) *	≤0.1 **	**ggTas2r7**
(+)-Catechin	0.3	3	**ggTas2r1, ggTas2r2, ggTas2r7**
QH	0.01	0.3 (Cheled-Shoval et al. [26])	**ggTas2r1**

In-vitro: thresholds determined by calcium imaging; in-vivo: thresholds determined by two-alternative forced choice (**2-AFC**) experiments. For values obtained from previous studies, the relevant references are listed. * Data for erythromycin in-vitro activation from Behrens et al. [7] were re-analyzed to provide in-vitro threshold of 0.1mM. ** Only 0.1 and 0.5 mM were examined and both were found to affect in-vivo behavior.

**Table 2:** List of Tested Compounds and Concentrations of the Tastant Solutions.

Tastant	Concentrations Tested (In-Vivo)	Concentrations Tested (In-Vitro)
**Nicotine**	0.1 mM, 0.33 mM, 1 mM, 3.3 mM, 10 mM	Behrens et al. [7]
**Caffeine**	33 µM, 0.1 mM, 0.33 mM, 1 mM, 3.3 mM, 10 mM	Behrens et al. [7]
**Erythromycin**	0.1 mM, 0.5 mM	Behrens et al. [7]
**(+)-Catechin**	33 µM, 3 mM, 1 mM	3 µM, 10 µM, 30 µM, 100 µM, 300 µM, 1000 µM
**QH**	Cheled-Shoval et al. [26]	0.1 µM, 0.3 µM, 1 µM, 3 µM, 10 µM, 30 µM

## Supplementary Materials

Supplementary materials are available online.
