# Supplementary material for: From Cell to Beak: In-Vitro and In-Vivo Characterization of Chicken Bitter Taste Thresholds
Source: Molecules. 2017 May 17;22(5):821. doi: 10.3390/molecules22050821 (PMC6154005; doi:10.3390/molecules22050821)
Supplement: Supplementary file 1 [file molecules-22-00821-s001.pdf]

Supplementary Data for Cheled-Shoval et al.

Functional Expression of Chicken Tas2r Constructs

Briefly, the coding region of the chicken receptors was fused to an N-terminal sst3-tag for efficient cell-surface targeting and to a C-terminal Herpes simplex virus (HSV) tag for immunolocalization. For the experiment, HEK 293Tcells stably expressing the G-protein chimera Ga16gust44 [1] were seeded onto poly-D-lysine-coated glass cover slips and incubated in DMEM containing 10% fetal bovine serum overnight at 37°C, 5% CO_2, 100% air humidity. The next day, cells were transiently transfected with the ggTas2r constructs using Lipofectamine 2000 according the manufacturer's protocol. As a negative control, an empty expression vector was included in the procedure. After additional ~24 h incubation, cells were washed twice for 1 min each in warm (37°C) 1X PBS and then placed on ice for 30 min. Next, biotinylated concanavalin A in 1X PBS was added at a dilution of 1:2000 and allowed to remain on the cells for 1h on ice. Five rinses for 1 min each with ice-cold 1X PBS were followed by fixation for 2 min in an ice-cold methanol–acetone (1:1, v/v) mixture. Cells were washed again for 1 min each with 1X PBS at room temperature. To block non-specific binding sites, cells were incubated for 45 min with 1X PBS containing 5% normal horse serum. Then, a mouse anti-HSV antibody was applied at a concentration of 1:15,000 in the same blocking buffer for 1 h at room temperature. After four 5-min washing steps with 1X PBS at room temperature, anti-mouse Alexa Fluor 488 antiserum (1:2000) and streptavidin Alexa Fluor 633 (1:100) in 1X PBS containing 5% normal horse serum were added to the cells for 1 h at room temperature. The cells were then washed three times for 5 min each with 1X PBS and incubated with 1:500-diluted DAPI in 1X PBS for 15 min at room temperature. Finally, the cells were rinsed three times for 5 min each with 1X PBS and once with ddH_2O before mounting on glass cover slips with Dako mounting medium for confocal microscopy.

Images were taken with a confocal laser-scanning microscope (Leica TCS SP8) at the following settings: for DAPI, 405nm excitation/440nm–460nm emission wavelength range for

detection; for Alexa Fluor 488,488nm excitation/510nm–540nm emission; for Alexa Fluor 633,633nm excitation/645–675nm emission.

For each transiently transfected receptor construct, three randomly chosen areas were scanned and used for cell counting. DAPI nuclear staining was monitored to assess the number of total cells, and the green fluorescent receptor-specific signals were monitored to evaluate the number of receptor-expressing cells. The cell counts of the three areas were averaged and the rate of expressing cells was given in percent ±SD.

Figure S1.

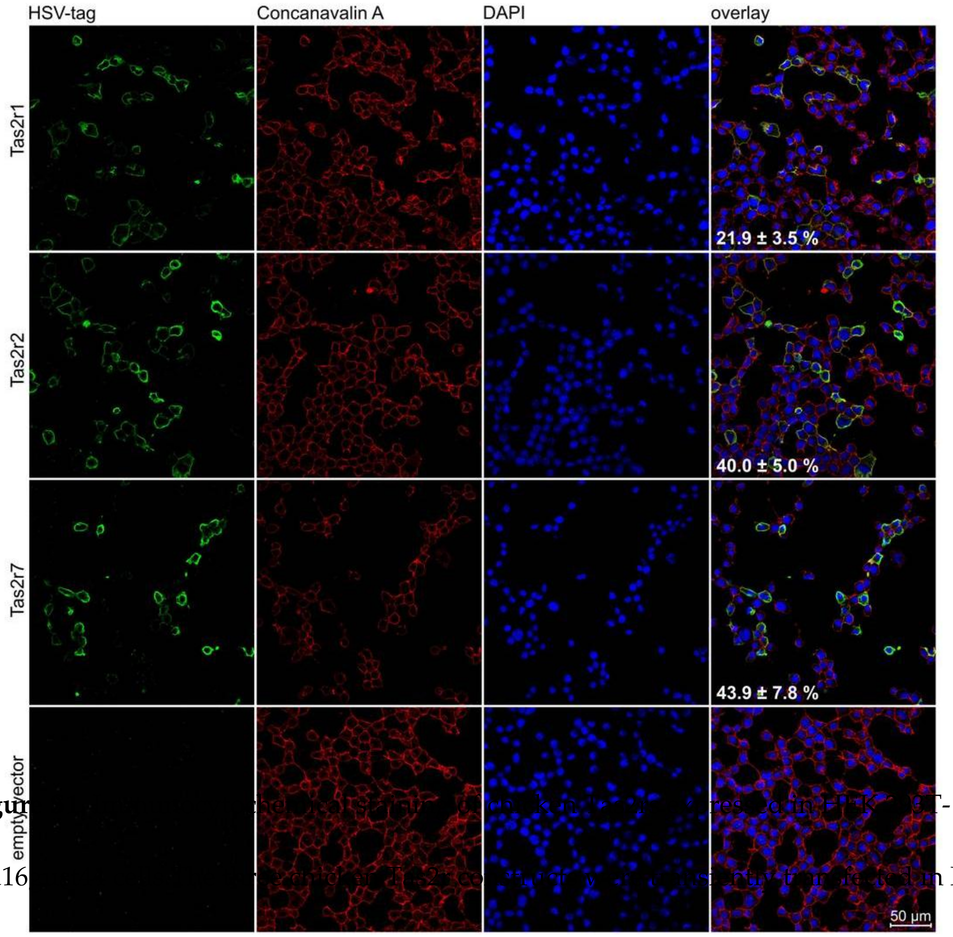

Figur[illegible] T-

Gα16[illegible] HEK 293T-Gα16gust44 cells. As a negative control, an empty expression vector was included. The receptor proteins were detected using an antiserum specific for the C-terminal-fused Herpes simplex virus glycoprotein D epitope (HSV-tag, green). The cell surface was labeled using concanavalin A (red) and the cell nuclei were stained with DAPI (blue). An overlay of the

three channels is shown in the right panels. Pictures were taken using a confocal laser-scanning microscope (Leica TCS SP8). For each chicken, Tas2r images of three randomly chosen areas were used to count the total number of cells (based on the number of DAPI-stained nuclei) and the number of receptor-positive cells (based on the number of green-labeled cells). The fraction of positive cells is shown in the overlay panels (% ± SD).

Figure S2.

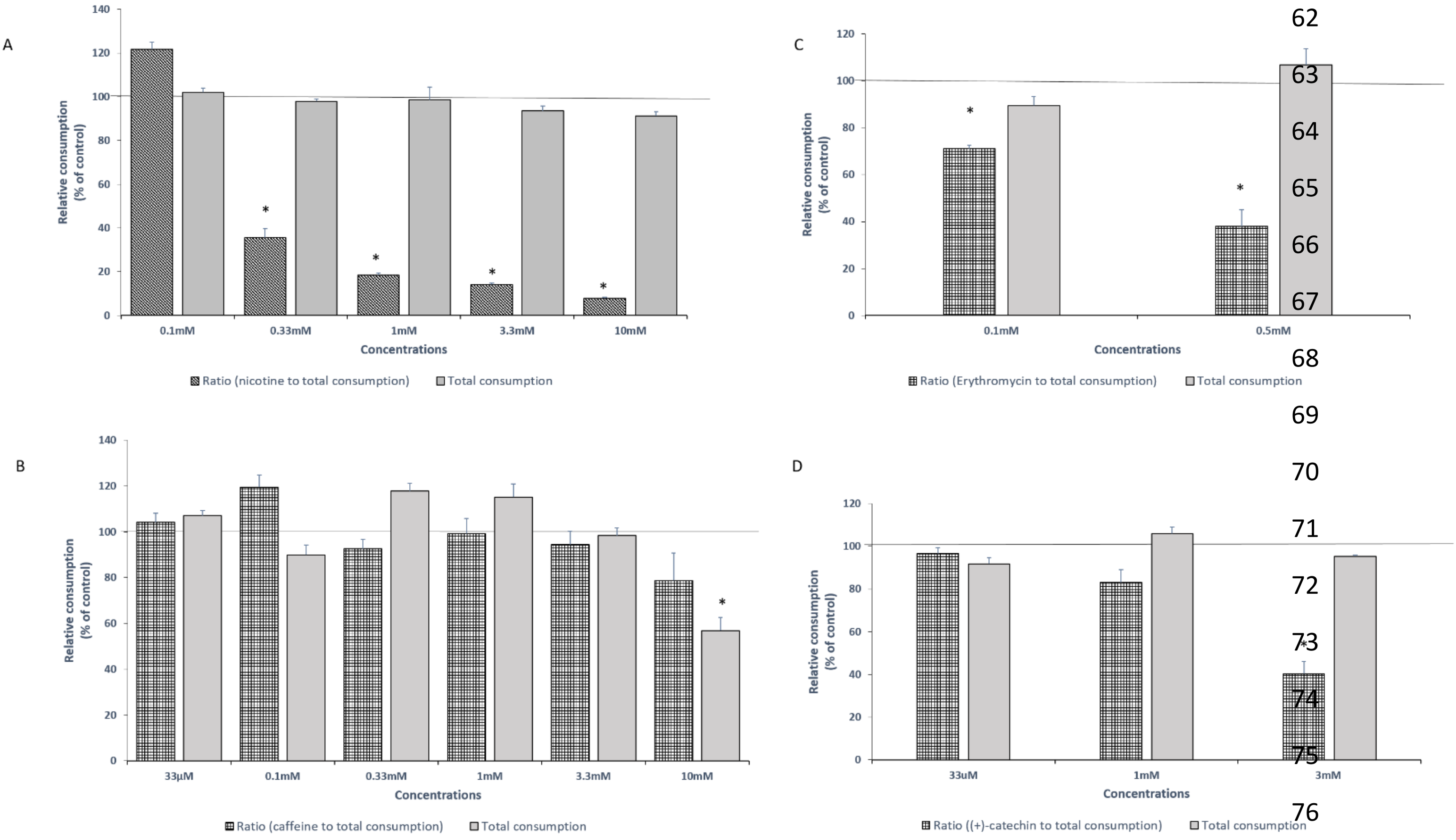

Figure S2. Effects of different concentrations of bitter tastants (A-Nicotine; B-Caffeine; C-Erythromycin; D-(+)-Catechin) on consumption parameters (per chick). Total consumption (tastant side and water side; per chick) and ratio (tastant consumption to total consumption) in the different concentrations (as percentage of control) after 24 h. All consumption parameters were normalized to the distilled water control group (=100%; indicated by black line at 100).

Bars represent consumption (represented as % of control group) ± SEM. * Significantly different from the control group at $P \leq 0.05$ by Dunnett's test.

Table S1. Primers used for real-time PCR analysis of mRNA abundance (Fold Change) calculation (All primers are as described in [2])

Gene name	Accession #	Forward primer (5`)	Reverse primer (3`)
ggTas2r1	AB249766.1	TTGAGTCAGTTGTGGGGCTT	GAAGTTGCTGTGTGCGTTGT
ggTas2r2	AB249767.1	GTCAACGGGGAACTGTGGAG	CCTCAATGCCAGTTTCAGCTT
ggTas2r7	NM_001080719	CTGTGCGCCACGTGGATATA	CCACAGGTTGGAAGAGCTTAAAA
Cyc A [3]	GQ849480.1	GGCTACAAGGGCTCCTGCTT	CCGTTGTGGCGCGTAAA
β-actin	NM_205518.1	AATGGCTCCGGTATGTGCAA	GGCCCATACCAACCATCACA

gg – Gallus gallus; Cyc A – cyclophilin A.

Supplementary References:

[1. Behrens, M.; Korsching, S.I.; Meyerhof, W. Tuning properties of avian and frog bitter taste receptors dynamically fit gene repertoire sizes. *Molecular biology and evolution* **2014**, *31*, 3216-3227.

2. Cheled-Shoval, S.L.; Druyan, S.; Uni, Z. Bitter, sweet and umami taste receptors and downstream signaling effectors: Expression in embryonic and growing chicken gastrointestinal tract. *Poultry science* **2015**, *94*, 1928-1941.

3. Van Herck, S.L.; Geysens, S.; Delbaere, J.; Tylzanowski, P.; Darras, V.M. Expression profile and thyroid hormone responsiveness of transporters and deiodinases in early embryonic chicken brain development. *Molecular and cellular endocrinology* **2012**, *349*, 289-297.
